# Trilobites in rock enrol: a comment on ‘Developmental and functional controls on enrolment in an ancient, extinct arthropod’ by Esteve and Hughes (2023)

**DOI:** 10.1098/rspb.2023.1547

**Published:** 2023-08-30

**Authors:** Ariel D. Chipman, Harriet B. Drage

**Affiliations:** ^1^ Department of Ecology, Evolution and Behavior, Silberman Institute of Life Sciences, The Hebrew University of Jerusalem, Edmond J. Safra Campus, Givat Ram, 91904 Jerusalem, Israel; ^2^ Institute of Earth Sciences, University of Lausanne, 1015 Lausanne, Switzerland

Roly-polies, woodlice, pillbugs, cheesy bugs, slaters … many names for one group of famous arthropods, the enrolling terrestrial isopods (figure 1*a*). These fascinating animals demonstrate a key behavioural innovation in the evolutionary history of arthropods: enrolment. In this behaviour, the individual curls its body into a ball to protect its vulnerable underneath (ventrum) and expose only the hardened dorsal exoskeleton. In some arthropods, enrolment behaviour is facilitated by specific morphological adaptations (coaptation), including ‘lock and key’ mechanisms in which there are structures along the body that interlock during enrolment and hold the individual within this enrolled posture. Enrolment is common in some extant arthropod groups, though overall rare, despite being crucial in providing effective protection from predation and parasitism. Not only does enrolment prevent predators and parasites from accessing the softer, vulnerable parts of an arthropod, but it also directs any additional protective exoskeletal structures, such as spines, outwards. Terrestrial isopods are the most famous examples of enrolment, though full enrolment is observed in scarab beetles (Ceratocanthini) [3], some millipede groups (figure 1*b*) [4] and various crustacean larvae (e.g. stomatopods, anomalans [5]), and partial enrolment (without encapsulating the entire ventrum) can also be seen in other myriapods (figure 1*c*) and beetles. The fossil record clearly shows that enrolment was adaptive in early arthropods during the Palaeozoic, and that this behaviour and its associated morphological developments have an extensive evolutionary history. Trilobites, hugely successful extinct marine arthropods from the Palaeozoic, demonstrate enrolment behaviours and related morphological adaptations. Several other extinct arthropod groups also show full or partial enrolment, including some xiphosurans, mollisoniids and potentially nektaspids (e.g. [6]). It is unclear why partial and perfect enrolment is rare in arthropods given its clearly adaptive role.

**Figure 1.**
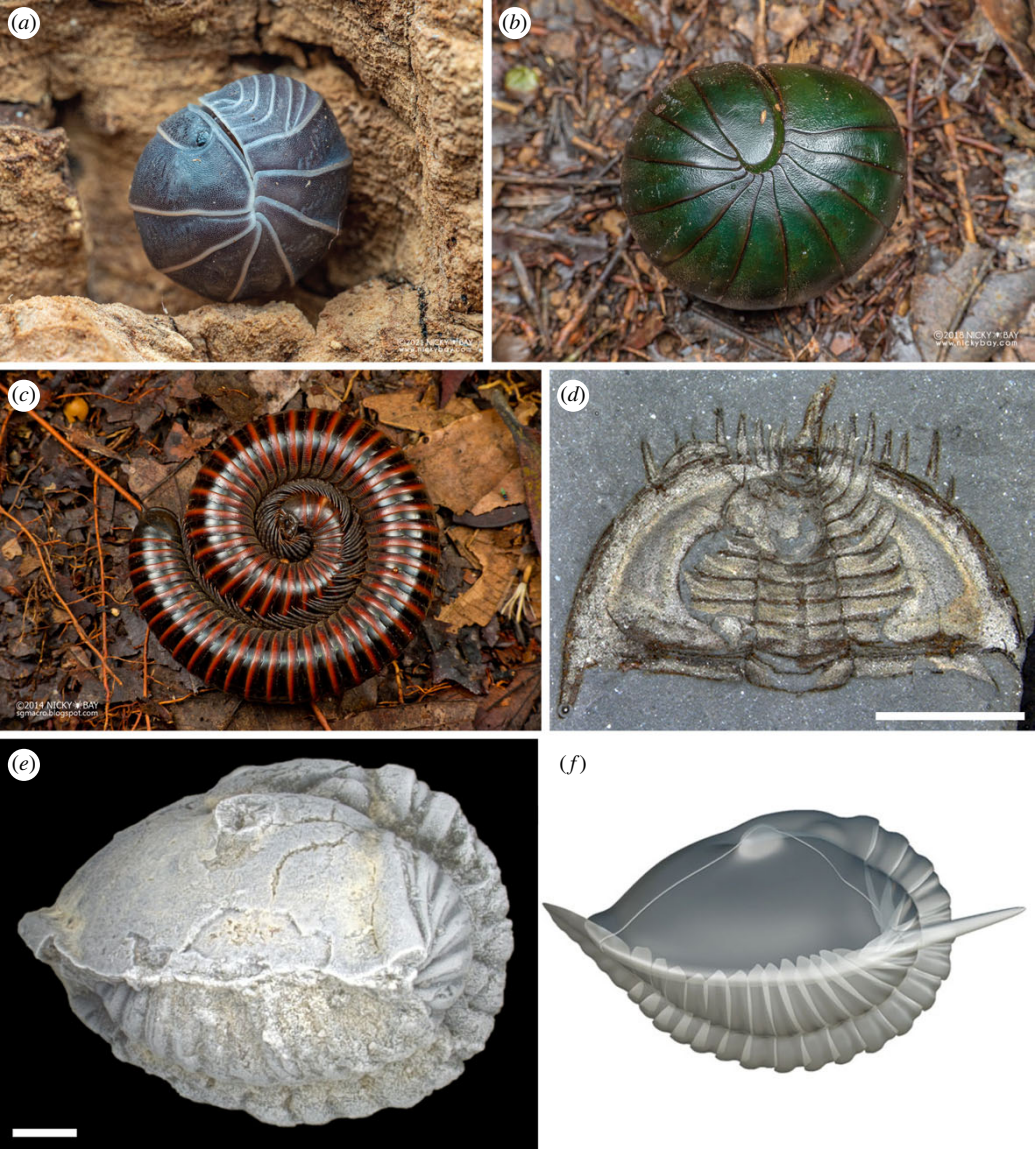
Examples of enrolling arthropods. (*a*) Completely enrolled *Armadillo officinalis* (Oniscidea, Isopoda) individual. (*b*) Completely enrolled giant pill millipede (Sphaerotheriida, Diplopoda) individual. (*c*) Incompletely enrolled millipede (non-pill millipede, Diplopoda). (*d*) Enrolled specimen of Early Cambrian *Mummaspis ?muralensis* (Olenellidae, Trilobita) demonstrating an incomplete enrolment style without coaptative mechanisms (specimen number GSC 137152). (*e*) Fully enrolled *Aulacopleura koninckii* (Aulacopleurida, Trilobita) individual used to produce three-dimensional enrolment models by Esteve & Hughes [1] (specimen number NMP-L12807). (*f*) Three-dimensional model created by Esteve & Hughes [1] showing hypothetical external spiral enrolment of adult *Aulacopleura koninckii* individual. Sources: (*a–c*) used with permission by the photographer, Nicky Bay (https://www.nickybay.com); (*d*) previously published by Ortega-Hernández *et al*. [2] and used with permission by the authors; (*e*,*f*) previously published by Esteve & Hughes [1] and used with permission by the authors. Scale bars = 5 mm (*d*), 1 mm (*e*).

Whether they enrol or not, arthropods face a crucial problem regarding growth due to their rigid exoskeletons. Before they can develop and grow, they must shed their exoskeleton in a process known as moulting. After a brief increase in size, usually achieved by inflating with air or water, they form a new exoskeleton befitting their increased size. This type of discontinuous growth might actually facilitate the maintenance of lock and key coaptative mechanisms, since every time a new exoskeleton is formed post-moulting, the different elements fit together from the start. By contrast, continuous growth requires constant coordination between the growth trajectories of different elements.

Arthropod growth becomes more complicated when it is accompanied by an increase in body length and in segment number. Many arthropods (most notably insects and almost all arachnids [7]) grow without an increase in the number of segments that comprise the body. Other groups (e.g. most millipedes and many crustaceans [7]) add segments during their development and growth, and these segments are always added during moulting. Segments may be added throughout the animal's life or only during the juvenile phase, and in any number of combinations of moulting and segment addition. For example, one segment per moult, one every other moult or several segments per moult. Glomerid millipedes (the familiar woodlands pill millipedes; figure 1*b*) represent an example of extant arthropods that are capable of enrolment while adding segments from moult to moult, at least during juvenile stages. Glomerids form perfectly enrolled balls, regardless of the number of body segments they possess. The exact mechanism for maintaining perfect enrolment despite these changes in segment number is unknown.

The fossil record of trilobites provides the most extensive, well-studied data regarding the evolution of enrolment behaviours and associated coaptative morphologies. Trilobites were a highly diverse and abundant animal group, with cosmopolitan distribution for much of the Palaeozoic (living *ca* 520–251 Ma), occupying all of the world's oceans and demonstrating the earliest innovations of many key arthropod behaviours, ecologies and life modes. They had strong biomineralized exoskeletons—mainly on the dorsal side—comprising a headshield (cephalon), multi-segmented thorax with segments attached by movable articulation structures, and a tailshield (pygidium). Trilobites were amongst the first enrolling arthropods, probably resulting from the need to protect the more weakly biomineralized ventrum, and, as such, are crucial to our understanding of this important behaviour. The earliest evidence of enrolment comes from the Early Cambrian, though we observe greater enrolment specialization from the Ordovician with the evolution of specialized coaptative structures that allow perfect enrolment (see [8] and references therein).

Incomplete (cylindrical) enrolment, which leaves gaps in the surface of the enrolled individual, has been described for the earliest groups of Cambrian trilobites, including the olenelloids, which had no coaptative structures and simply bent the trunk segments to curl around themselves (figure 1*d*) [2]. Successive groups show a variety of enrolment styles, including the common sphaeroidal type with the pygidium resting beneath the cephalon, and lateral pleurae interlocking, as well as several other types with the cephalon and pygidium variously over/underlapping [8]. Trilobites clearly demonstrate the evolution of specialized enrolment through their evolutionary history; the number of coaptative devices increased during the Ordovician, which may be associated with increased cephalon morphological disparity [2]. For example, some groups, such as Middle Cambrian solenopleuropsines [9] and phacopines from the Silurian and Devonian [10], had a vincular furrow and notches, which allowed the pygidium to lock into place with the cephalon. Many trilobite groups developed spectacular exoskeletal spinosity, including genal (posteriorly directed cephalic spines), cephalic, pygidial and axial (along the central lobe of the body) spines, which would have greatly aided defence while enrolled in any style. While enrolment was and is clearly important for defensive purposes in arthropods, partial enrolment (i.e. flexing of the body segments) also appears to have been used to facilitate moulting in trilobites [11]. Interestingly, moulting-related partial enrolment has been described, like presumed defensive enrolment, from the Early Cambrian [12], though it would be almost impossible to determine for which end enrolment first originated. Given the multipurpose utility of enrolment, it is possible that development of the trilobite exoskeleton was restricted by the need to maintain enrolment postures, and so this behaviour, particularly full defensive enrolment, was presumably intrinsically linked to ontogeny in the group.

A new perspective on trilobite enrolment is provided by a recent paper by Esteve & Hughes [1]. The paper gives a detailed description, based on biomechanical modelling, of how the trilobite *Aulacopleura koninckii* might have enrolled. Esteve & Hughes base their modelling on well-preserved fossil material from the Silurian of Czechia, representing an ontogenetic series for almost the entire life cycle of the species. While almost all specimens in the series are preserved in an outstretched (unenrolled) position, a small number of specimens are preserved intact and with almost no compression in fully enrolled postures (figure 1*e*). The authors use these fossilized examples of enrolled individuals as the basis for reconstructing three-dimensional models of enrolled trilobites at all ontogenetic stages (figure 1*f*). It is worth noting that uncompressed fossils of enrolled trilobites are fairly rare, even in a rich assemblage like that providing the Czech *A. koninckii* specimens, and the three-dimensional models thus provide a detailed perspective that is almost never seen in the fossil record.

The ontogeny and enrolment behaviour of *A. koninckii* is particularly interesting due to the species's variability in thoracic segment number during the adult phase. As in all trilobites, segments are added sub-terminally in successive moults and are gradually released from the pygidium to the thorax. Segment addition usually halts when the animal reaches the adult stages, and successive moults only facilitate exoskeleton repair and general growth. However, in *A. koninckii* the final thoracic segment number in adults varies from 18 to 22 segments, and the species therefore has five different adult morphotypes. Esteve & Hughes [1] ask a number of pertinent questions regarding this developmental variability. During juvenile stages, perfect sphaeroidal enrolment is achieved regardless of the number of thoracic segments. However, the variable number of thoracic segments in the adult stage necessitates a transition in enrolment style. Sphaeroidal enrolment that perfectly encapsulates the ventral body cannot work for the different body lengths and articulation points in adult forms.

Esteve & Hughes [1] suggest a number of different solutions to this segment number constraint, and implement them in their biomechanical model. The simplest option is for the adult *A. koninckii* to continue attempting sphaeroidal enrolment. However, the model shows that this type of enrolment would leave vulnerable gaps in the ventral exoskeleton and is therefore not viable. The second option is for *A. koninckii* to employ spiral enrolment, with the tip of the pygidium tucked under the cephalon (internal spiral enrolment, in the authors’ terminology). This type of enrolment still leaves gaps, though not as significant as those resulting from sphaeroidal enrolment. More significantly, internal spiral enrolment requires one or more segment articulations to flex at a much sharper angle than all other articulation joints; an unreasonable assumption given the presumed consistency in morphology and putative musculature between the different thoracic segments. The remaining option is external spiral enrolment, in which the pygidium overlies the cephalon. This type of enrolment is determined to be the most reasonable biomechanical option for adult *A. koninckii* individuals based on segment articulation angles and pygidium placement, and is consistent with the situation found in a number of, albeit poorly preserved, fossil specimens.

The modelled change in enrolment style at the onset of adulthood in *A. koninckii* also leads Esteve & Hughes [1] to discuss several conundrums regarding trilobite enrolment, pathology and moulting. Interestingly, teratological expression of segment articulation, where trunk segments remained partially fused to each other and were thus unable to flex during enrolment, was found only in adult morphotypes of *A. koninckii*, and was strikingly common at approximately 10% of specimens. This is of particular interest, because such thoracic articulation teratologies are generally rare in trilobites [13], probably due to their inherent interference with movement and protective behaviours like enrolment, leading to the premature death of affected individuals. It seems that the different enrolment style in *A. koninckii* individuals with more trunk segments may have been resistant to this articulation teratology, or indeed the (large) adults themselves were less likely to be predated, reducing the need for perfect enrolment and causing this teratology to be less strongly selected against. Future work focusing on a potential link between articulation teratologies and the specific morphotypes and body sizes impacted might shed light on the reason for the presence of the teratologies. Esteve & Hughes [1] also observed coincident timing between this change in enrolment style in adults with an apparent change in moulting behaviour, at a similar developmental timing to that determined for the Czech trilobites *Dalmanitina proaeva* and *Dalmanitina socialis* [14], providing further evidence for an association between enrolment and moulting that would be worth broader exploration.

While the beautiful biomechanical models presented by Esteve & Hughes [1] provide an appealing and convincing answer to the question of how trilobites enrolled, they also leave the reader with a number of intriguing questions. How is the ventral side of the pygidium protected in an individual during external spiral enrolment? Does the pygidium of *A. koninckii* fit tightly enough to the cephalon without coaptative mechanisms? Are the muscles that facilitate enrolment variable among thoracic segments, or is the difference in the angle of flexion between segments only a function of the enrolment posture? Were adults with segment teratologies simply too large to be impacted by imperfect enrolment? These questions might remain unanswered, but the dynamic images of ancient animals enrolling leave us rocking and rolling.

## Data Availability

This article has no additional data.
